# A Contemporary Review of Plasminogen Activator Inhibitor Type 1: Structure, Function, Genetic Architecture, and Intracellular/Extracellular Roles

**DOI:** 10.1055/a-2698-4219

**Published:** 2025-09-26

**Authors:** Jacob Wortley, Justin Vu, Neha Soogoor, Mebeli Becerra, Mohanakrishnan Sathyamoorthy

**Affiliations:** 1 Sathyamoorthy Laboratory, Department of Medicine, Burnett School of Medicine at TCU, Fort Worth, Texas, United States; 2 Department of Cardiovascular Medicine and Science, Fort Worth Institute for Molecular Medicine and Genomics Research, Fort Worth, Texas, United States

**Keywords:** PAI-1, extracellular matrix, genetics, fibrinolytic system, cell migration, cell proliferation

## Abstract

Plasminogen activator inhibitor type 1 (PAI-1) is the key regulator of the fibrinolytic system, thereby acting as a potent mediator in thrombosis. Plasminogen activators such as PAI-1 mediate the conversion of the inactive zymogen plasminogen to plasmin, an active serine protease. As a member of the serpin superfamily, the highly conserved structure of PAI-1 is critical for its regulatory function. This review elucidates PAI-1 structure, function, and genetic architecture, and then discusses intracellular and extracellular functions that have broad implications for proliferative signaling and cell death, angiogenesis, cellular transit, and emerging roles in cancer biology. By understanding the complex and elaborate mechanism of PAI-1 in the fibrinolytic system and as a biomarker, PAI-1 may have broad implications across many disease states not related to its historical roles in fibrinolysis and thrombosis.

## The Fibrinolytic System

The human fibrinolytic system plays a pivotal role in hemostasis and thrombosis related to multiple organ systems. At the heart of the proteolytic cascade lie serine proteases—tissue-type plasminogen activator (tPA) and urokinase-type plasminogen activator (uPA)—responsible for catalyzing the conversion of the zymogen, plasminogen, into the active form, plasmin, through the cleavage of a serine–arginine to valine peptide bond. 1 Plasmin, a key player in the fibrinolytic system, directly catalyzes the degradation of the fibrin protein meshwork that results in the dissolution of thrombi. While tPA primarily facilitates intravascular fibrinolysis through localization of plasminogen and fibrin deposits on the endothelium, uPA is observed predominantly in the pericellular environment, regulating localized proteolysis critical for cell invasion, wound healing, and tumor metastasis. 2 3 Plasminogen activator inhibitor type 1 (PAI-1), a potent serine protease inhibitor (serpine), plays a significant regulatory role for uPA/tPA to establish an equilibrium between plasminogen and plasmin as well as maintaining a stable pericellular environment for optimal cellular function. Thus, dysregulation of PAI-1 expression leads to multiple downstream pathologies that contribute to coagulopathy and other systemic disease states such as cardiovascular disease, metabolic syndrome, and cancer. 4 Numerous other disease processes include inflammation and infection, diabetes, and neurodegeneration, with PAI-1 associated with aging, skeletal muscle repair, and cellular senescence. PAI-1's role in the fibrinolytic system has clinical relevance, with the excess or deficiency that results in bleeding disorders. Specifically, hyperfibrinolytic disorders can present as bleeding of unknown origin but are largely reported in patients with a PAI-1 deficiency, suggesting a greater need for specific diagnostic testing and timeliness for treatment. 5

To better recognize the role of PAI-1 as a regulatory checkpoint, it is critical to understand the mechanism of its substrate. uPA is synthesized as an inactive, single-chain zymogen (pro-uPA) composed of 411 amino acids distributed across distinct functional domains: A growth factor domain (GFD; residues 1–49), a Kringle domain (residues 50–131), an interdomain connecting peptide (residues 132–158), and a serine protease domain (residues 150–411). 6 7 Activation occurs through a single cleavage event at Lys158–Ile159, resulting in a two-chain, active enzyme stabilized by a disulfide bond between Cys148 and Cys279. 8 9 Subsequent proteolysis can further process uPA, separating the amino-terminal fragment (residues 1–135)—responsible for receptor binding and signaling—from the enzymatic serine protease domain. 8 9

The effects of uPA are spatially restricted and amplified through its interactions with its high-affinity receptor, urokinase plasminogen activator receptor (uPAR), or CD87. 10 Belonging to the Ly-6/uPAR superfamily, mature uPAR consists of 283 residues derived from a 335-residue precursor after subsequent signal peptide removal and the addition of a glycosylphosphatidylinositol moiety. 10 11 Structurally, uPAR is organized into three homologous Ly-6/uPAR/α-neurotoxin-like domains—D1, D2, and D3—which form a central hydrophobic cavity that interacts with the GFD of uPA, inducing conformational changes at the D1–D2 interface that primes the receptor's affinity for secondary ligands, notably vitronectin (VN), a highly abundant protein found in the subendothelial matrix. 11 12 13 14 Crystal structural studies reveal that while uPA occupies the hydrophobic core of uPAR, VN associates externally through its somatomedin domain (SBD; residues 1–44), enabling simultaneous ligand engagement. 12 Binding of uPA shifts uPAR into a closed, high-affinity conformation for VN and initiates intracellular signaling cascades—such as focal adhesion kinase (FAK), the Janus (JAK) and Src family kinases, and the phosphatidylinositol 3-kinase/protein kinase-B (PI3K/Akt) axis—that ultimately culminates in enhanced cytoskeletal remodeling, increased motility, or greater survival signaling. 11 15

**Fig. 1:**
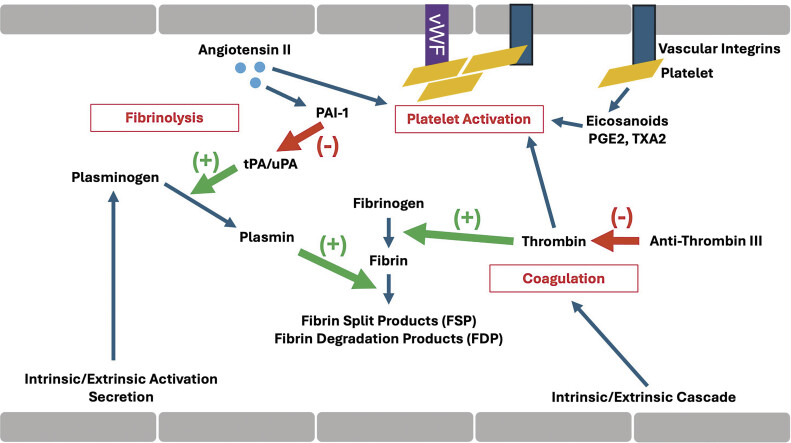
Overview of the cell-based model of thrombosis and central role for PAI-1 in regulation of fibrinolysis. PAI-1, plasminogen activator inhibitor type 1.

Given the central role of uPA/uPAR interactions in coordinating cellular processes implicated in the pathogenesis of disease progression, the need for precise regulation is critical, and thus, has garnered the attention of many researchers and clinicians as a potential target to inhibit. Consequently, much of the research has been focused on PAI-1, a key endogenous regulator of uPA that is a part of the serine protease inhibitor superfamily known as serpins. Interestingly, the most peculiar is the bevy of recent literature that has implicated PAI-1 in exerting both promigratory and antimigratory effects independent of its conventionally associated functions within proteolysis. This paradox highlights the complex interplay among these tightly regulated interactions, underscoring perhaps a delicate balance within the cellular microenvironment and the physiological or pathological processes involved. This review aims to summarize our current knowledge on the diverse cellular functions of PAI-1 that have broad implications for emerging roles in cancer biology and therapeutics ( Fig. 1 ).

## Plasminogen Activator Inhibitor Type 1

### Structure, Stability, and Regulation

**Fig. 2:**
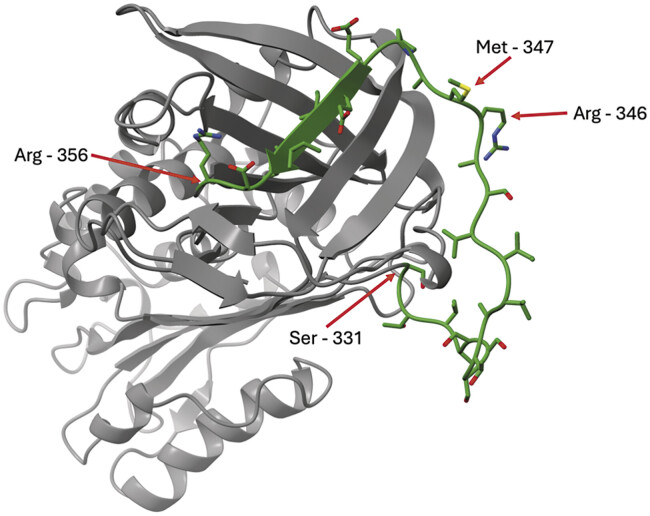
Protein structure of plasminogen activator inhibitor type 1 with reactive center loop highlighted and labeled residues. Molecular graphics and analyses performed with University of California, San Francisco (UCSF) ChimeraX, developed by the Resource for Biocomputing, Visualization, and Informatics at the University of California, San Francisco, with support from the National Institutes of Health R01-GM129325 and the Office of Cyber Infrastructure and Computational Biology, National Institute of Allergy and Infectious Diseases. 18

Glycoprotein PAI-1 is a 45-kDa, 379- to 381-amino acid protein expressed within several tissue beds with a primary role in regulating the fibrinolytic pathway. Being in the serpin superfamily, PAI-1 exhibits a highly conserved structure seen in other serine proteases. Specifically, PAI-1 contains the characteristic three β-sheets (A, B, and C) as well as nine α-helices (hA–hI) that are typical of this family of proteases. Critical for its function, a reactive center loop (RCL) composed of 26 residues (331-SGTVASSSTAVIVSARMAPEEIIMDR-356) is critical to its function as a plasminogen inhibitor ( Fig. 2 ). 16 17 18 This sequence is particularly important at Arg346 and Met347 (P1-P1′) due to its role as a substrate mimic toward tPA and uPA. The remainder of the RCL sequence is thus referred to as P16-P10′ regarding its position around these key residues. Importantly, the P16-P10′ region confers specificity to the individual proteins within the serpine family.

The remainder of the amino acids outside this region are highly conserved among this superfamily of proteins, and mutagenic studies have confirmed that alteration of the RCL changes its function. Specifically, studies indicate that mutations in the P1-P1′ region can alter the structure to act as antithrombin III depending on the alterations made to the base sequence. 19 20 21 As a target-specific region, PAI-1 selectively inhibits plasminogen activators (PAs) to perform its role as an antifibrinolytic agent.

PAI-1 is highly expressed in many tissues, such as the liver, respiratory system, kidneys, placenta, vascular smooth muscle cells, endothelial cells, and adipose tissue. Once secreted into the circulation, it exists in an active conformation for only a few minutes (plasma *t*_1/2_ 6 minutes) before being rapidly cleared by the liver. 22 Structurally, PAI-1 exists in two forms, active and latent, which modulate its activity toward PA in a conformationally dependent manner. Once PAI-1 is secreted by endothelial cells, it exists in two main reservoirs intravascularly—free plasma, and retained in circulating platelets. The free plasma concentration is predominantly in an active conformation but only accounts for as much as 5 to 50 ng/mL in serum. 23 Conversely, platelet retained PAI-1 accounts for much higher levels up to 300 ng/mL but exhibits mostly latent conformation. 23 Interestingly, research has indicated that platelet activation induced by endothelial damage shows a significant increase in the active form of PAI-1. This presents a benefit to reinforcing clot formation at the onset of damage to ensure proper hemodynamic stability during times of injury.

**Fig. 3:**
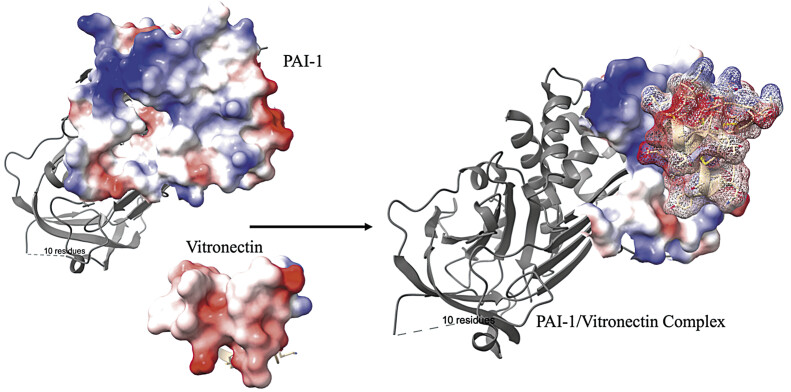
Protein structure of PAI-1 and somatomedin B domain of vitronectin with resultant depiction of protein–protein binding of PAI-1/vitronectin complex. Coloring of the protein surface indicates polarity of acidic (red), alkaline (blue), and uncharged (white) side chains. Vitronectin is composed of relatively acidic R groups that interact strongly with PAI-1's alkaline-binding pocket. 18 PAI-1, plasminogen activator inhibitor type 1.

PAI-1 exerts its effects in a tissue-specific manner due to glycosylation at Asn209 and Asn265 and its interaction with local mediators like PA. 23 Inhibitory members of the serpin family, such as PAI-1, interact through the formation of a covalent 1:1 stoichiometric complex with their target proteinases. 24 Formation of an ester bond between the carboxyl group of the P1 residue of the serpin and the hydroxyl group of the active serine residue of the target protease inhibits the activity of the target protease. 24 The crystal structure of latent PAI-1 was originally elucidated by Mottonen et al in 1992, followed by determination of the crystal structure of active PAI-1 by Nar et al. 25 26 Structure–function experimentation has revealed that the major, biologically relevant difference between latent and active forms of PAI-1 is that active PAI-1 has an exposed bait region (P1-P1′; Fig. 2 ) known as an RCL (R346-M347), as well as accessible secondary binding sites that enable inhibitory complex formation with a target protease. In latent PAI-1, the bait region and secondary structures are inaccessible and cannot bind to the active site of serine proteases. 23 The active conformation of PAI-1 in human tissue is maintained by complexing with VN, a highly abundant protein found in the subendothelial matrix. It is likely that VN-bound PAI-1 ( Fig. 3 ) is the physiologically relevant mechanism for maintaining the inhibitory function of PAI-1 in human tissue. Importantly, the structural characteristics of PAI-1 present key interactions that can be leveraged during the development of effective therapeutics discussed later in this review.

Recognizing the importance of the RCL to PAI-1's function, it is critical to elucidate the mechanisms surrounding this inhibitory process of PAs. Specifically, two major pathways exist once RCL interacts with the PA active site: The irreversible inhibitory pathway and the substrate pathway. Prior to entering a specific sequence, the RCL chain first binds to PA, forming a weak, non-covalent Michaelis complex. 17 23 27 28 This step is followed by attack of the PA active site serine (tPA-Ser478 or uPA195) onto the RCL to produce an unstable tetrahedral intermediate with PAI-1. 17 29 The tetrahedral intermediate undergoes subsequent collapse that forms a more stable acyl–enzyme intermediate with resultant covalently bound PAI-1/PA complex. 30 31 From the acyl–enzyme complex, PAI-1 activity induces either the irreversible inhibitory or substrate pathway. Importantly, the inhibitory sequence accounts for most PAI-1 interactions and follows a complex rearrangement of three-dimensional structure. Crystal structure analysis indicates that the high-energy state of acyl-intermediate formation encourages protein rearrangement to a lower-energy conformation that results in shifting of the P16-P1 portion (N-terminal side) into the core β-sheet of PAI-1. 29 The thermodynamically driven rearrangement ultimately fuels the compression of PA against the core of PAI-1, leading to effective blocking of its active site. 32 Resultant deformation of PA thus prevents hydrolysis of the PAI-1/PA complex and yields an irreversible inhibition of function. 30 31 Conversely, reports have identified an alternative substrate pathway that effectively regenerates PA after the formation of the acyl–enzyme intermediate. It is proposed that hydrolysis occurs prior to PA deformation in the core of PAI-1, which releases PA before the P16-P1 region and can undergo conformational change. 17 23 33 34 It is thought that a distinct subset of PAI-1 in this alternative substrate pathway exists or can be induced through local intermediaries or kinetic properties, which could precipitate more active PA. 35 36 37 38

Interestingly, PAI-1 regulation centers on deactivating the RCL through conformational change from the active-to-latent transition. Studies have shown that the in vitro half-life of the active conformer of PAI-1 is around 2 hours and occurs through a slow, self-insertion of the N-terminal region of the RCL into the core protein. 17 26 This insertion functionally blocks the P1-P1′ region from interacting with PAs such as tPA and uPA. It is hypothesized that this form of autoregulation presents an ancestral benefit in reducing the risk of thrombosis due to otherwise extended antifibrinolytic activity of PAI-1. 16 17 Additionally, in vivo analysis of PAI-1 stability has been extensively studied and relies upon interaction with the ubiquitous plasma glycoprotein, VN. VN, as discussed throughout this review, serves as a cofactor for PAI-1 and stabilizes its active form, approximately twofold, through stabilization of the lower half of its protein structure without affecting enzymatic activity to regulate PA. 39 40 41 42 This stabilization ultimately slows the conversion to latent-type PAI-1 and serves as an important barrier to keep in mind during the development of therapeutic targets.

### Genetic Architecture

**Fig. 4:**
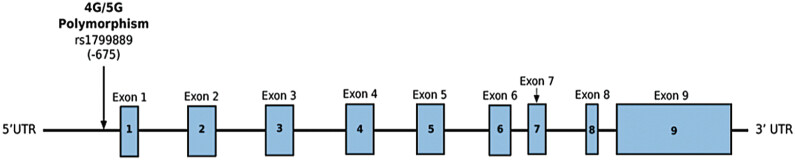
Schematic representation of the *SERPINE1* gene structure. The *SERPINE1* gene, encoding plasminogen activator inhibitor type 1, is located on chromosome 7q21.3-q22 and spans approximately 12.3 kb. The gene consists of nine exons (blue boxes) and eight introns (connecting lines). The 4G/5G polymorphism is shown at −675 bases upstream of the transcriptional start site.

PAI-1 is encoded by the *SERPINE1* gene, located on chromosome 7q21.3-q22 (NCBI accession number M16006). This gene spans approximately 12.3 kb and consists of nine exons and eight introns, as shown ( Fig. 4 ). 22 43 44 45 46 47 48 49 50 51 Two distinct mRNA transcripts, 2.3 and 3.2 kb in length, are produced from this gene due to alternative polyadenylation, with the longer transcript potentially exhibiting greater stability due to an AT-rich sequence. 52

**Fig. 5:**
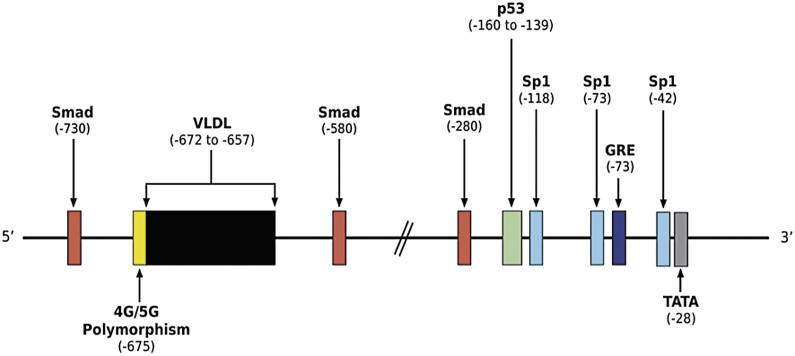
Schematic representation of the 5′ promoter region of the *PAI-1* gene and its regulatory elements. The PAI-1 promoter contains multiple transcriptional regulatory elements influencing gene expression. The −675 4G/5G polymorphism (yellow) is associated with higher expression. A VLDL response element (−672 to −657, black) links PAI-1 expression to lipid metabolism. Smad-binding elements (red) at −730, −580, and −280 indicate TGF-β/Smad pathway regulation. A p53-responsive element (−160 to −139, green) suggests a role in cellular stress responses and tumorigenesis. Sp1-binding sites (−118, −73, −42, light blue) mediate glucose-induced upregulation. A glucocorticoid response element (GRE; −73, dark blue) suggests modulation by glucocorticoids. The TATA box (−28, gray) is essential for transcription initiation. PAI-1, plasminogen activator inhibitor type 1.

The 5′ promoter region of the *PAI-1* gene is a complex landscape of regulatory elements with significant implications in cardiovascular disease ( Fig. 5 ). 43 44 52 In the presence of wound healing, p38 mitogen-activated protein kinase (MAPK) is rapidly activated in the MAP kinase pathways in cells adjacent to the injury, leading to phosphorylation of the transcription factor upstream stimulatory factor-1 (USF-1), a member of the helix–loop–helix family. This phosphorylated USF-1 complex then translocates to the nucleus, where it binds to the E-box motif (CACGTG sequence located at nucleotides −160 to −165) in the proximal promoter region of the *PAI-1* gene; this binding is essential for upregulation of PAI-1 transcription. Notably, activator protein-1 (AP-1)-binding sites do not play a significant role in the PAI-1 regulation model, with the MAPK–USF-1 axis the primary pathway linking mechanical injury to PAI-1 gene activation. 53 This pathway is not only active in wound healing, but is a critical player in vascular injury response and cardiovascular disease. 47 53 During vascular injury due to shearing, inflammation, and diabetes, the MAPK–USF-1 pathway is activated as described above, promoting the upregulation of PAI-1 transcription. Additionally, the presence of a glucocorticoid response element site enables upregulation of PAI-1 expression by glucocorticoids like dexamethasone and mineralocorticoids like aldosterone. 43 54 55 This is particularly relevant in hypertension, where angiotensin II stimulates PAI-1 expression and release, which can be further amplified by glucocorticoids and aldosterone. 43 54 56 A VLDL response element situated within the PAI-1 promoter region at positions −672 to −657 facilitates interaction with very low density lipoprotein (VLDL), a lipoprotein often elevated in diabetes and metabolic syndrome, suggesting potential clinical implications for these conditions. 43 57 58 Sp1 sites in the promoter region are positively regulated by glucose and glucosamine, linking elevated glucose levels to increased PAI-1 levels, and potentially contributing to the development of diabetes. 43 59 60 Additionally, the presence of Smad-binding element and p53-responsive element in the PAI-1 promoter suggests potential involvement in various cancers. 43 61 62

Another important aspect of the PAI-1 promoter is the −675 4G/5G polymorphism. This insertion/deletion polymorphism affects PAI-1 levels, with the 4G allele associated with higher levels than the 5G allele. 43 63 64 65 Although the exact clinical consequences of this polymorphism are under debate, its association with various diseases, including stroke and venous thromboembolism, emphasizes the significance of PAI-1 in a wide range of clinical conditions. 43 66 Collectively, these conserved regulatory elements provide insights into the mechanisms that modulate PAI-1 levels and function in vivo and influence various clinical conditions ranging from cardiovascular diseases and metabolic disorders to cancer. Understanding these regulatory mechanisms enhances our grasp of PAI-1's role as both a biomarker and a potential therapeutic target across different disease states characterized by thrombotic risk and altered fibrinolysis.

### Intracellular and Extracellular Functions

It is extensively characterized that the uPA/uPAR system plays a central role in tightly regulating extracellular matrix (ECM) remodeling essential for supporting cell migration. Disruption of this balance can have pathological consequences. 11 67 68 69 Excessive ECM degradation leads to compromised structural integrity and enhanced cell invasion, while insufficient matrix turnover results in abnormal ECM accumulation and impaired cellular dynamics. 9 68 Early studies substantiate that PAI-1 critically modulates ECM dynamics by interacting with multiple extracellular components, including PAs, VN, and low-density lipoprotein receptor-related protein 1 (LRP1). 70 71 72 73 Through these interactions, PAI-1 has been shown to both inhibit and promote cell migration depending on the contextual molecular environment. 68 71 74

### Regulation of Plasmin Generation

One of the major functions of PAI-1 is its ability to modulate coagulation through its interaction with tPA and uPA. By blocking tPA/uPA agonist activity on the conversion of plasminogen to plasmin, PAI-1 effectively inhibits fibrinolysis through a deficiency of plasmin levels. 75 In the absence of PAI-1, uPA bound to uPAR and tPA/fibrin activates plasminogen through cleavage of Arg561–Val562 residues of each proenzyme. 75 This cleavage results in the generation of plasmin, which binds to fibrin to produce fibrin degradation products. Mechanistically, PAI-1's binding to uPA/tPA (as described above) leads to the collapse of the tPA and uPA active site for plasminogen. Therefore, plasminogen is unable to effectively bind and undergo cleavage into plasmin products. Interestingly, research on patients with homozygous PAI-1 deficiency exhibited increased peak plasmin levels and resulted in hyperfibrinolytic bleeding. 76 Due to its effective inhibition of tPA/uPA as well as its rapid onset of action when released, PAI-1 remains a critical component of the balance between coagulation and fibrinolysis.

### Plasminogen Activator Inhibitor Type 1 and Vitronectin: A Central Player in Cell Migration through Urokinase Plasminogen Activator Receptor

VN is established to play a pivotal role in this process. The somatomedin B domain of VN binds to PAI-1, stabilizing its active conformation and enhancing its inhibitory effect on uPA-dependent proteolysis, thereby reducing ECM degradation and limiting the cellular ability to migrate and invade. 77 78 Furthermore, PAI-1 exhibits a stronger binding affinity to the SMB domain of VN compared with uPAR. 71 During binding of the somatomedin B domain of VN, PAI-1 structurally distorts the arginine-glycine-aspartate (RGD) sequence of VN, the primary binding site for αvβ3 integrins, affecting the formation and turnover of focal adhesions, essential for cell migration. 24 69 This competitive binding is a key mechanism through which PAI-1 indirectly modulates integrin function without directly binding to the integrins themselves. 72 As a result, elevated levels of PAI-1 can lead to detachment of cells from VN-rich matrices by blocking integrin-mediated adhesion, promoting cell motility or dysmotility in certain contexts. 72

PAI-1 also contributes to cell signaling through its effects on uPAR–integrin associations. The binding of uPA to uPAR promotes the assembly of uPAR–integrin complexes, which in turn facilitates the activation of FAK and Src family kinases through caveolin-mediated clustering at the cell surface. 9 15 These complexes are necessary for initiating adhesion-dependent signal transduction. 9 15 Disruption of uPAR–integrin interactions by PAI-1, through VN competition, impairs this signaling cascade. 79 80 Notably, excessive uPAR activity has also been shown to drive extracellular-signal related kinase (ERK) activation and exit from tumor dormancy by promoting α5β1-dependent FAK/Src signaling. 81 Thus, PAI-1-mediated interference with uPAR–integrin clustering may serve as a regulatory checkpoint in these pathways.

Furthermore, PAI-1 indirectly influences G-protein-coupled receptor signaling via its control over uPAR cleavage. When uPA binds to uPAR or when the D1 domain is proteolytically removed, a chemotactic epitope (Ser88–Tyr92) becomes exposed, allowing the receptor to engage G-protein coupled receptors (GPCRs) such as formyl peptide receptor-like 1. 82 83 84 85 86 87 These interactions promote cytoskeletal rearrangement and cell movement in a pertussis toxin-sensitive manner. 88 By modulating uPAR availability and surface retention, PAI-1 can indirectly regulate the activation of these chemotactic pathways.

Experimental studies have further confirmed the relevance of PAI-1–integrin dynamics to cell movement. Treatment of cells with recombinant PAI-1 mimics the effects of αvβ5 integrin blockade, resulting in cell detachment from VN and enhanced migration toward other ECM proteins such as fibronectin or collagen type IV. 72 79 89 These findings highlight PAI-1's ability to remodel adhesion patterns by interfering with integrin–ECM engagement, independent of its classical role as a serine protease inhibitor. Further investigations by Sathyamoorthy et al 90 employed RNA interference to inhibit PAI-1 expression in HL-60 human monocytes. Transfection of small interfering RNAs targeting PAI-1 mRNA achieved an 85% reduction in PAI-1 mRNA and complete inhibition of glycosylated PAI-1 protein expression after 72 hours. 90 This posttranscriptional silencing revealed a significant increase in monocyte adhesion attributable to enhanced interactions between VN and its receptors. Further investigations by Sathyamoorthy et al 91 demonstrated the role of PAI-1 in endothelial progenitor cell (EPC) regulation. Using bone marrow-derived EPCs from wild-type and PAI-1-deficient mice, they found that PAI-1 deficiency led to a 75% increase in early outgrowth EPCs and enhanced proliferative capacity. 91 Similarly, in a model of chronic vascular injury using N()- nitro-L-arginine methyl ester (L-NAME), PAI-1-deficient mice showed a 65% increase in circulating EPCs in comparison to c57BL6 wild-type controls. 91 These results suggest that PAI-1 deficiency can enhance EPC proliferation and mobilization, potentially offering protective effects against vascular injury.

Conversely, PAI-1 has also been reported to promote cell migration by protecting the ECM against excessive plasmin-mediated degradation, providing a provisional matrix that tumor and endothelial cells use as a scaffold for migration or capillary formation. 68 92 In fact, a recent study by McCann et al demonstrated that a decrease in TGF-β signaling caused a decrease in miR-30 expression, which subsequently resulted in the upregulation of PAI-1, decreased uPA activity, and fibrin-mediated angiogenesis. 93 Restoration experiments in PAI-1-deficient mice confirmed that reintroducing recombinant PAI-1—particularly functional at the VN-binding site—could restore tumor angiogenesis, further highlighting the dual requirement for matrix preservation and controlled proteolysis during new vessel formation. 94 95 Collectively, these studies underscore the context-dependent nature of PAI-1 in cell migration, suggesting that the relative stoichiometry of PAI-1, VN, and uPA, as well as the other factors in the cell microenvironment, significantly influences these processes.

### Plasminogen Activator Inhibitor Type 1 and Low-Density Lipoprotein Receptor-Related Protein 1-Mediated Endocytosis and Integrin Regulation

Beyond its interaction with VN, PAI-1 can influence cell migration through its interaction with LRP1, a member of the LDL receptor family. 96 The low-density lipoprotein receptor-related protein family is involved in numerous physiological processes such as regulating cholesterol homeostasis and lipid transport, supporting brain development and function, facilitating nutrient and vitamin transport, aiding the uptake of essential molecules, and participating in signal transduction. 97

Following uPA/PAI's detachment from VN, the cryptic receptor-binding site of PAI-1 becomes exposed, allowing the molecule to bind to LRP1. 59 60 61 This interaction initiates endocytosis of the uPA/uPAR/PAI-1 complex along with associated integrins. Inside the cell, PAI-1 and uPA are targeted for lysosomal degradation, while uPAR and LRP1 are recycled back to the plasma membrane, particularly at the leading edge of migrating cells. 9 98 99 100 This recycling process enables cells to establish new adhesion points in different ECM regions, contributing to persistent migration across tissue substrates. Notably, while LRP1-mediated integrin endocytosis can occur, it is not strictly required for PAI-1-initiated cell detachment, suggesting that PAI-1 can promote cell detachment through multiple mechanisms, not solely dependent on LRP1-mediated integrin internalization. 98 Despite losing their antiprotease activity, latent or cleaved forms of PAI-1 retain the capacity to bind to LRP1 independently and promote cell migration. 96 101 102 These forms can, therefore, remain embedded in the ECM, potentially serving as a reservoir for active PAI-1 to maintain cell movement. 103

LRP1 plays a dual role in endocytosis and signal transduction. PAI-1 facilitates the internalization of uPA/uPAR complexes by binding LRP1 via its cryptic receptor-binding domain exposed after detachment from VN. 68 104 In doing so, PAI-1 regulates receptor trafficking while also modulating intracellular signaling. Studies have shown that LRP1 suppresses Rac1 and ERK activation in an uPAR-dependent manner. 73 96 105 In LRP1-deficient mouse embryonic fibroblasts or in cells treated with receptor-associated protein to block LRP1, Rac1 activity, and cell motility increase—an effect absent from uPAR-knockout cells, confirming the requirement of uPAR for this pathway. 106 By controlling LRP1-mediated trafficking, PAI-1 thus influences Rho GTPase-driven cytoskeletal dynamics and downstream MAPK activation, which in turn leads to cytoskeletal rearrangements, including the formation of lamellipodia and filopodia, which are essential for cell motility.

PAI-1 also plays a role in modulating intracellular signaling through the JAK/STAT pathway, albeit indirectly, by regulating the availability and clustering of the uPA/uPAR complex at the cell surface. When uPA binds uPAR, receptor clustering can recruit JAK1 and activate STAT1, leading to STAT1 dimerization, nuclear translocation, and transcription of target genes via interferon-γ activation site and interferon-stimulated response elements. 74 This signaling is facilitated by co-localization with gp130. 74 107 By inhibiting uPA binding and promoting internalization of uPA/uPAR complexes through LRP1, PAI-1 reduces surface receptor density and attenuates JAK/STAT activation. These effects have been observed in epithelial and vascular smooth muscle cells where PAI-1 influences migration and gene expression through upstream control of this pathway. 108 109

The intricate interplay between the components of the uPA system, particularly the multifaceted role of PAI-1, emphasizes the complexity of cell migration regulation. PAI-1's ability to influence cell behavior through interactions with uPA, its receptor uPAR, LRP1, integrins, and VN highlights its significance as a potential therapeutic target in diseases characterized by dysregulated cell migration and ECM remodelling. While PAI-1 inhibition of uPA-mediated proteolysis can suppress cell migration, its interactions with other molecules like VN and LRP1 can promote cell detachment and signaling, leading to increased motility. This dual nature of PAI-1 explains the paradoxical observations in clinical settings, which have been observed to be associated with high levels of PAI-1 with poor prognosis in specific cancer patient populations. 110 111 112

### Sustaining Proliferative Signaling

Building on its established role in ECM regulation and modulation of uPA/uPAR signaling, PAI-1 also contributes to proliferative signaling in cancer by shaping the extracellular and pericellular environments that control receptor activation. While some studies have shown PAI-1 to inhibit proliferation in certain cancer types, like hepatocellular carcinoma, 92 ovarian, 113 and prostate cancer, 92 most research indicates that PAI-1 promotes cancer cell proliferation through various mechanisms. 114 115 Giacoia et al demonstrated that PAI-1 downregulation inhibited cell proliferation by inducing G0–G1 cell cycle arrest, whereas PAI-1 overexpression enhanced S-phase entry and increased tumor size in xenograft models. 116 This is consistent with findings by Mashiko et al, who demonstrated that PAI-1 knockdown or inhibition blocked ovarian cancer cell proliferation through G2–M cell cycle arrest and intrinsic apoptosis. 117 Similarly, Li et al found that PAI-1 acts as a tumor enhancer in aggressive fibromatosis but reduces tumor cell proliferation and motility when deficient. 118

These proliferative effects are largely mediated through PAI-1's indirect influence on surface receptor availability and proteolytic microenvironment. By inhibiting uPA- and tPA-mediated plasminogen activation, PAI-1 limits plasmin generation and maintains thrombin activity in the tumor microenvironment. Elevated thrombin levels can activate protease-activated receptors (PARs), particularly PAR-1 and PAR-2, which are G-protein-coupled receptors implicated in tumor cell proliferation, migration, and angiogenesis. 119 120 121 Thrombin-induced PAR-1 activation triggers intracellular signaling via MAPK/ERK and PI3K/Akt pathways, linking PAI-1-mediated fibrinolytic suppression to enhanced mitogenic responses. Furthermore, PAI-1 fibronectin-dependent cell growth by regulating cell detachment from VN and adhesion to fibronectin. 120 121 PAI-1 also modulates growth factor receptor signaling by affecting uPA/uPAR complex dynamics. Stabilization or clearance of these complexes can influence epidermal growth factor receptor (EGFR) activation, as well as ERK and AKT signaling, as observed in lung fibroblasts. 122 In other contexts, such as neuronal cells, PAI-1 enhances nerve growth factor-mediated responses by maintaining the proteolytic balance necessary for receptor engagement. 123

Despite these growth-promoting roles, PAI-1 can also induce senescence or dormancy under stress or in the presence of p53 activation. 124 125 126 PAI-1 is a well-established transcriptional target of p53, and its upregulation is essential for enforcing p53-mediated replicative senescence. Kortlever et al demonstrated that PAI-1 expression alone was sufficient to induce a senescent phenotype in fibroblast cells even in the absence of functional p53, permitting cells to bypass senescence. 124 Mechanistically, PAI-1 has been shown to inhibit proteasome activity, resulting in stabilization of p53 and sustained activation of the p53–p21–pRb axis, thereby enforcing irreversible cell cycle arrest. 124 Aguirre-Ghiso et al showed that PAI-1 inhibition of uPA/uPAR activity can induce tumor cell dormancy by altering the ERK/p38 activity ratio. 127 Kubala and DeClerck 92 highlighted this ongoing controversy, stating that while some studies 116 show that PAI-1 stimulates cell cycle progression through cyclin D3/CDK4/6 upregulation, other studies 128 indicate PAI-1 has an inhibitory function on proliferation in breast cancer cell lines. Thus, without a precise molecular mechanism identified, the direct impact of PAI-1 on tumor cell cycles remains a subject of debate.

### Resisting Cell Death

PAI-1 contributes to tumor cell survival by modulating apoptosis through both indirect protease inhibition and direct intracellular signaling mechanisms. PAI-1 has been shown to suppress both extrinsic and intrinsic apoptotic pathways. 129 Extrinsically, it protects cells from Fas-mediated apoptosis by regulating pericellular plasmin activity, thereby preventing the cleavage and shedding of Fas ligand (FasL) in various human cancer cell lines. 129 130 131 This function is primarily mediated through PAI-1's ability to inhibit uPA and maintain low plasmin levels, thereby preventing proteolytic activation of proapoptotic pathways.

Intrinsically, PAI-1 has been shown to interact with and inhibit caspase-3, a key executioner of apoptosis, as demonstrated in vascular smooth muscle cells and endothelial models. 129 132 133 This inhibition may occur through a cross-class serpin mechanism involving the RCL of PAI-1, although this interaction remains incompletely defined. Furthermore, PAI-1 has been shown to upregulate antiapoptotic proteins, such as B-cell lymphoma 2 (Bcl-2) and B-cell lymphoma-extra large (Bcl-xL), while downregulating proapoptotic proteins, including Bcl-2-associated X protein (Bax) and B-cell lymphoma-extra small (Bcl-xS). 129 132 133

These effects are mediated primarily through activation of the PI3K/Akt and ERK signaling pathways. By promoting the internalization of uPA/uPAR complexes via LRP1, PAI-1 activates PI3K, which phosphorylates and activates Akt. 9 134 Activated Akt enhances p53 transcriptional activity, suppressing transcription of proapoptotic genes such as *Bax* while also enhancing the stability and expression of antiapoptotic proteins, Bcl-2 and Bcl-xL. Akt also phosphorylates and inactivates Bad, preventing it from displacing Bcl-2 from its inhibitory complexes. Simultaneously, ERK activation downstream of LRP1 signaling may contribute to transcription of Bcl-2 family genes, supporting cell survival in stress conditions. These pathways have been observed in endothelial and vascular smooth muscle cells in which PAI-1 expression correlates with resistance to serum starvation or detachment-induced apoptosis. 135 136 Notably, the antiapoptotic activity of PAI-1 can be attenuated using neutralizing antibodies, as demonstrated by Balsara et al, who accelerate its conformational inactivation, suggesting that only the active form of PAI-1 possesses this prosurvival function. 137

Despite this, several studies indicate that PAI-1's role in apoptosis is not uniformly protective. Depending on the tumor type and microenvironment, PAI-1 overexpression has been associated with increased apoptosis and reduced tumor growth. 138 139 140 As seen in certain breast and colon cancer models, PAI-1 overexpression or transfection enhanced apoptotic sensitivity. 130 141 This functional duality underscores the complexity of PAI-1's role in cancer biology. These multifaceted roles make PAI-1 an important determinant of tumor cell fate and a compelling candidate for targeted therapy in apoptosis-resistant cancers.

### Angiogenesis

PAI-1 plays a complex, dose-dependent role in tumor angiogenesis by regulating endothelial cell migration, matrix remodeling, and protease activity through both direct and indirect mechanisms. 95 140 142 At physiological concentrations, PAI-1 exhibits proangiogenic activity through multiple mechanisms. It inhibits FasL cleavage by plasmin, preventing Fas-mediated apoptosis in endothelial cells. 143 One mechanism involves inhibition of plasmin-mediated cleavage of FasL, thereby protecting endothelial cells from Fas-dependent apoptosis and allowing sustained angiogenic activity. 94 130 In parallel, PAI-1 facilitates endothelial migration by disrupting adhesion to VN—through competitive binding at the somatomedin B domain—and promoting movement toward fibronectin-rich areas typical of tumor stroma. 130 144

By inhibiting plasminogen activation, PAI-1 contributes to fibrin accumulation, which can enhance endothelial organization and the release of proangiogenic cytokines such as interleukin-8. 145 146 This matrix stabilization not only provides a scaffold for new vessel formation but also creates a localized environment favorable to cell guidance and alignment during neovascular sprouting. 144 Moreover, PAI-1 promotes interleukin-8-mediated neutrophil transendothelial migration by preventing the proteolytic shedding of IL-8/heparan sulfate/syndecan-1 complexes. By stabilizing IL-8 on the endothelial surface, PAI-1 enhances IL-8 bioavailability and augments its proangiogenic signaling effects. 147 These functions underscore PAI-1's essential role in coordinating immune cell-driven and matrix-dependent processes that support angiogenic remodeling.

Importantly, VEGF-induced activation of uPA on endothelial cells leads to the formation of uPA/PAI-1 complexes on the surface, which are then internalized via LRP1. This internalization is required for targeted recycling of uPAR to the leading edge, a process essential for directional migration during sprouting angiogenesis. 74 The recycling of uPAR was significantly impaired in PAI-1-deficient cells, further underscoring PAI-1's non-proteolytic role in orchestrating the migratory behavior of endothelial cells via receptor trafficking. 74

Conversely, at supraphysiological concentrations, PAI-1 inhibits angiogenesis by suppressing uPA and plasmin activity, both of which are required for matrix degradation and endothelial invasion. Bajou et al showed in PAI-1 knockout models that normal physiological levels of host-derived PAI-1 are essential for effective tumor vascularization, whereas excessive PAI-1 impairs this process by overly restricting proteolysis. 140 This biphasic behavior explains the paradox wherein PAI-1 is associated with aggressive tumor progression despite its protease-inhibiting function, reflecting its role in maintaining the proteolytic balance needed for controlled angiogenesis. Notably, the cellular source of PAI-1 is a critical determinant of its angiogenic function. Host-derived PAI-1 at physiological concentrations promotes tumor invasion and angiogenesis; whereas, PAI-1 produced by tumor cells, even at high concentrations, cannot compensate for its absence in the host. 140 Transgenic mice overexpressing PAI-1 in tumor cells or tumor cell lines transfected with PAI-1 cDNA exhibited impaired vascularization, suggesting that excessive or localized PAI-1 disrupts the spatial and temporal proteolysis needed for neovessel formation. 140 148 This suggests that PAI-1's angiogenic function depends not only on its concentration but also on its cellular origin and its ability to regulate endothelial receptor localization, inflammatory signaling, and matrix composition.

## Conclusion

Aside from PAI's critical role in the fibrinolytic system as the key natural proteolytic regulator of tPA in plasma, PAI-1 also exhibits numerous intracellular and extracellular functions. PAI-1 is a central player in cell migration and ECM remodeling through the uPA/uPAR system through interaction with multiple extracellular components, including PAs, VN, and LRP1. Given the strong link between uPA–uPAR pathway and PAI-1, with its impact on cellular migration, the need for precise regulation is critical and a potential area for future research as a target for inhibition through small molecules, RNA aptamers, monoclonal antibodies, and repurposed pharmaceuticals.

Additionally, PAI-1 has a significant role in functions including endocytosis, integrin regulation, proliferative signaling, angiogenesis, and invasion and metastasis. Notably, PAI-1 has been shown to have pro- and anticarcinogenic effects, including cell cycle progression through both extrinsic and intrinsic apoptotic pathways, along with cellular senescence and dormancy, highlighting the emerging role of PAI-1 in cancer biology. Even though PAI-1's impact on tumor cell cycles remains a subject of debate, future research can be centered around PAI-1 as a compelling candidate for targeted therapy in apoptosis-resistant cancers. As such, this review advances the understanding of PAI-1's diverse roles in cellular biology and its growing importance in numerous health and human disease states spanning cardiovascular disease to oncology beyond its well-understood historical associations in thrombosis. Our group further highlights PAI's role in neurodegenerative diseases, metabolic disorders, and aging in our subsequent contemporary review of this literature review series.
